# PD118 Company-Led Submissions For Cancer Medicines: The Singapore Experience

**DOI:** 10.1017/S0266462324003581

**Published:** 2025-01-07

**Authors:** Jiamin Ong, Lydia Loke, Liang Lin, Kwong Ng

## Abstract

**Introduction:**

The Agency for Care Effectiveness (ACE) conducts health technology assessments (HTAs) to inform funding decisions by the Ministry of Health (MOH) Drug Advisory Committee (DAC) in Singapore. In 2021 ACE introduced the company-led submission (CLS) process for cancer medicines, which allows pharmaceutical companies to request evaluations alongside regulatory reviews. This review reports key findings from the first year of its implementation.

**Methods:**

A total of 10 CLS topics from the first year of implementation were included. We reviewed the status and outcomes of the DAC recommendations. We also used descriptive statistical methods to evaluate the time from HTA submission to first HTA recommendation and from regulatory approval to first HTA recommendation. The timelines were further analyzed by whether submissions were parallel submissions (i.e., HTA submission in tandem with regulatory review) or sequential submissions (i.e., HTA submission after regulatory approval). These statistics were compared with overseas reference jurisdictions (Australia, Canada, and the UK).

**Results:**

At the time of review, three topics were pending discussion. Of the remaining seven topics, three (43%) received positive recommendations for inclusion on the MOH Cancer Drug List and three (43%) received negative recommendations. The DAC was unable to make a recommendation on one topic. The median time from HTA submission or regulatory approval to first HTA recommendation was 172 days (range 169 to 263 days) and 279 days (range 53 to 374 days), respectively. Notably, parallel submissions (75 days; n=2) had considerably shorter timelines from regulatory approval to first HTA recommendation than sequential submissions (328 days; n=4). These timelines were within the range of the overseas reference countries.

**Conclusions:**

Parallel CLS allows HTA processes to be conducted in tandem with regulatory reviews, moving HTA recommendations upstream and expediting patient access to clinically effective and cost-effective medicines. Efforts will be made to further evolve the CLS process to achieve timely reimbursement reviews from regulatory approval and to expand this process to noncancer medicines.

